# Emerging Strategies and Applications of Layer-by-Layer Self-Assembly

**DOI:** 10.5772/60009

**Published:** 2014-01-01

**Authors:** Deepak Rawtani, Yadvendra K. Agrawal

**Affiliations:** 1 Gujarat Forensic Sciences University, Sector 18A, Near Police Bhawan, Gandhinagar, Gujarat, India

**Keywords:** Self-assembly, Fuel Cells, Biosensor, Langmuir-Blodgett, H_2_ sensing

## Abstract

Layer-by-layer self-assembly is an approach to develop an ultrathin film on solid support by alternate exposure to positive and negative species with spontaneous deposition of the oppositely charged ions. This paper summarizes various approaches used for fabrication of layer-by-layer self-assembly as well as their utility to produce various devices. The layer-by-layer technique is basically used for formation of multilayer films. A variety of nanomaterials use it for the modification of films to enhance their resultant durability as well as strength. Studies have shown that many different types of materials can be used for fabrication of multilayers. Recently the layer-by-layer self-assembly technique has also been used for fabrication of gas sensors, hydrogen sensors and solar-based cells. Various methods, such as spin deposition, calcinations, and dry-transfer printing are being used for fabrication of thin films. In this review, the author summarizes the various interesting properties as well as fabrication strategies of layer-by-layer self-assembly.

## 1. Introduction

Layer-by-layer self-assembly is a technique used to grow an ultrathin film on solid substrates by ‘flip-flop’ or alternate exposure to positive and negative species with instantaneous deposition of the oppositely charged ions [1]. Since the discovery of Langmuir-Blodgett (LB) phenomena for adsorption of different charged species by thin films, this technique is being extensively employed for development of multilayer architectures with controllable thickness. The technique generates multilayers with highly ordered nanoscale features, which depend on the type of organic material used [2, 3]. Subsequently, alternative assemblies of oppositely charged colloids on glass support and sequentially layered substrates with oppositely charged metal ions carrying polycrystalline coatings were originated [4, 5]. Besides being simple and robust, these methodologies require minimally sophisticated technology. By application of precise stoichiometry, they can be optimized easily and do not depend on complicated chemical reactions to deposit successive layers. Recently, the layer-by-layer self-assembly approach has emerged as a real alternative to the Langmuir-Blodgett technique. Electrostatic forces are the main driving forces for layer-by-layer self-assembly, but at times hydrogen-bond interaction is involved as well. Layer-by-layer self-assembly is an emerging discipline of nanotechnology in which objects, devices and various systems with varying structures are formed without externally applied prodding. Layer-by-layer self-assembly is basically a thin-film fabrication approach, which involves deposition of opposite charges containing polyions for the formation of alternating layers with concomitant washing steps in between.

### 1.1 Substrates for layer-by-layer self-assembly

**Figure 1.**
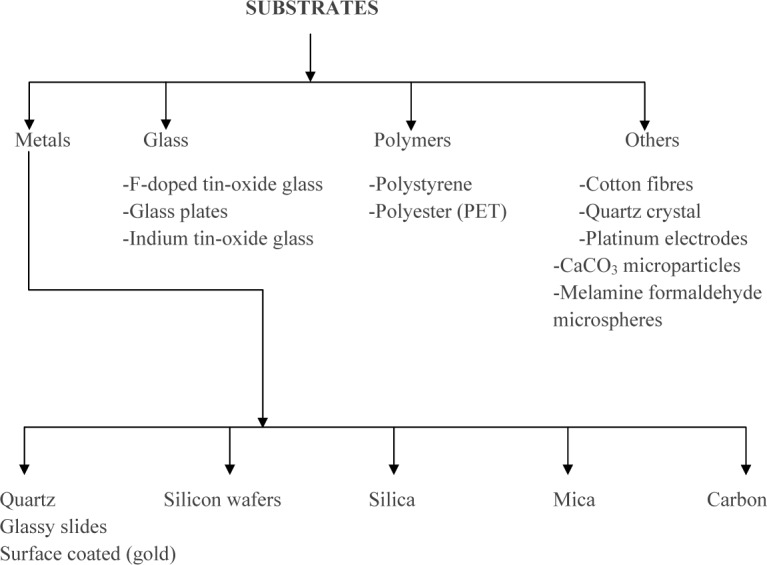
Classification of substrates used in fabrication of layer-by-layer self-assembly

The most important requirement for layer-by-layer self-assembly is a suitable substrate which can hold as well as support the assembly that is going to be organized on it (Table 1). A variety of different substrates are used to create different assemblies, including glass, quartz, silicon wafers, mica and different polymers (Fig. 1).

**Table 1.** The various substrates utilized in the layer-by-layer self-assembly technique and their applications

S.N. Type of application	Substrate
1. Microbial fuel cells	Carbon Toray Paper
2. Ampheteric biosensors	Platinum electrode
3. Bio-based urushiol-Ti ultrathin film with anticorrosive property	CaF_2_ plate, quartz slide, Cu sheet
4. Humidity sensors	Polyimide
5. Gas sensors	Quartz crystal
6. Bio-film inhibition	Titanium
7. Petroleum refinery waste-water treatment	Poly(ethyleneimine) (PEI)/titania (TiO2)
8. Stent-assisted gene transfer	Gold
9. Self-assembly of anionic and cationic CNT	Polymer
10. Nanoparticle coating on lignocelluloses wood microfibres	Kraft softwood fibres
11. DNA-dye complex film by self-assembly	Quartz
12. Mercaptosulphonic-acid-capped silver nanoparticles	Quartz
13. Dye-sensitized solar cells	Polymers PAA (poly[acrylic acid]) PAH (poly[allylamine hydrochloride])
14. H_2_ gas sensing	Polyester (PET)
15. Silicate coating of Yb_2_O_3_ and SiO_2_ particles	Silicon
16. Increasing solubility of CNTs in water	Polymer
17. Amperometric glucose biosensor using Prussian blue (PB)	Negatively charged ITO/PB electrode
18. Hydrazine phosphorus thin film containing dendrimers	Silicon wafers, gold-coated glass slides
19. Magnetic cantilever arrays with 2D micro patterns and 3D SWNTs	Silicon and polymer
20. Synthesis of anti-reflection thin films	Glass, polystyrene and Si/SiO_2_
21. Strength and durability of MEMS	Poly(allylamine hydrochloride), poly (acrylamino acid)
22. UV protection for cotton fabrics	Cotton substrates
23. Synthesis of ultrathin organicmultilayer films using squarylium dye	Glass
24. Photo-luminescent PAMAM-CdS nanocomposites	Melamine formaldehyde (MF) microspheres

Layer-by-layer self-assembly can be instituted on a large number of substrates. They are as follows:

## 2. Strategies for different kinds of multilayers using layer-by-layer self-assembly

### 2.1 Fuel-cells preparation

Microbial fuel cells (MFCs) are specially designed devices which convert chemical energy of fuel into electrical energy via catalytic action of electrogenic anaerobic microorganisms [6-8]. The material used for fabrication of electrodes has a great influence on the resultant power-generation capacity. The applied material enhances the accessible surface area for bacteria to anchor and subsequently affects the interfacial electron transfer and resistance. Therefore it is crucial to select an appropriate material for fabrication of electrodes to modify the resultant anode surface with an electro-active species, in order to facilitate uninterrupted and continuous electron transfer. Due to the chemical stability and efficient biocapacity of carbon Toray Paper (TP) electrodes, they are very commonly used for these applications. For this, conc. H_2_SO_4_-HNO_3_ is applied a priori to the TP electrode in order to activate the surface with an initial charge (negative charge of carboxyl groups). A layer-by-layer self-assembly is formed by dousing the negatively charged TP electrode in a positively charged aqueous solution of PEI (polyethyleneimine). To further balance the positive charges these electrodes are then immersed in aqueous solution containing negatively charged multi-walled nanotubes (MWNT). This procedure allows us to fabricate thin film with minimum effort. Furthermore, unabsorbed materials can be washed off by immersing the dipped electrode into ultrapure water in between subsequent dipping steps.

Similar methodology can also be applied for the formation of amperometric glucose biosensors based on the nine layers of multilayer films using multi-walled carbon nanotubes, glucose oxidase (GOD – extracted from species of Aspergillus Niger) and gold nanoparticles. Carbon nanotubes have unique mechanical, electrical and chemical properties besides having enormous potential for use as an important component of nanoscale electronic devices. Due to efficiency at higher temperatures (high thermal capacity) and promotion of higher electron transfer, CNTs are an important component of biosensors [9, 10].

### 2.2 Amperometric-biosensors preparation

A novel strategy to fabricate amperometric biosensors is found in multilayer thin films via layer-by-layer self-assembly of a gold nanoparticles (GNp)/multi-walled carbon nanotubes hybrid and glucose oxidase (GOD) for the analysis of glucose. A prerequisite for the fabrication of biosensors is the immobilization of enzymes on the immobilization support in order to achieve efficient surface immobilization of the enzyme on the surface of the electrode. The hybrid of nanoparticles and nanotubes was prepared by the immobilization of gold nanoparticles on multi-walled carbon nanotubes (MWCNTs). Usually the self-assembly process is carried out on a pre-treated platinum electrode dipped in a solution of polyallyamine (PAA) for surface activation and followed by subsequent washing with buffer saline. At a later stage, the number of layers to be deposited decides the number of dips into different solutions. Several washing steps also follow in between each dipping. Eventually, layers formed on the multilayer thin films comprise the GOD/GNP/MWCNTs/Pt electrode.

In another fabrication strategy, glucose-based biosensors were developed. In this approach, immobilization of glucose oxidase (GOD) was executed for the resultant fabrication of Prussian blue (PB) glucose biosensors using chitosan and multi-walled nanotubes (MWNTs) as a support. Principally, the process for the fabrication of biosensors involves the adsorption of enzymes or electrolytes from a solution onto the surface of an electrode, either through electrostatic forces or covalent bonding [11]. Chitosan has an excellent film-forming capability as well as a magnificent ability to act as a polymeric scaffold for enzyme immobilization [12–13]. Chitosan combines with carbon due to its poor conductivity. Besides having higher stability, Prussian blue (PB) exhibits high electro-catalytic activity and high selectivity for electro-reduction of H_2_O_2_ at low employed potential, and is thus used for H_2_O_2_ detection and biosensor fabrication [14-16].

In the fabrication of these types of biosensor, Prussian blue heightens the sensitivity of biosensors to detect glucose even at low potential. Amperometric glucose-based biosensors are fabricated using multilayer films of chitosan/MWNT/GOD. A negatively charged indium-tin-oxide (ITO)/PB electrode is used as a support on which chitosan, MWNTs and GOD are absorbed alternately by electrostatic interaction with subsequent washing and nitrogen drying in between. The resultant assembly of chitosan/MWNT/GOD is repeated until the desired number of layers is attained (6–7). The concomitant biosensor has several advantages, such as high selectivity, lower detection limit, improved sensitivity and higher affinity for glucose, as well as good stability under optimal conditions [17].

### 2.3 Dendrimer-based molecular thin films

More and more interest is being shown in applications of nanotechnology. Dendrimers are being used as building blocks for the fabrication of molecularly thin multilayer films on the basis of electrostatic layer-by-layer self-assembly [18-30]. Dendrimers have long been used for interdisciplinary research, for example in catalysis, optoelectronics, photo-physical processes, and encapsulation of guest molecules [31-44]. Various studies pertaining to biomedical development especially related to applications of dendrimers are combined with polyelectrolytic multilayers (PEMs) [45, 46]. Recently, multilayer thin films composed of hydrazine phosphorus containing dendrimers or dendrimers with linear polystyrene sulphonate (PSS) were prepared by the layer-by-layer self-deposition approach. The resultant polyelectrolyte multilayers (PEMs) containing dendrimer were studied for their potential as bioactive surfaces as well as for control of protein/cell adhesion [47]. Various substrates, such as polished silicon wafers and gold-imbibed glass slides have been used as support materials for dendrimer-based polyelectrolyte multilayers. The behaviour of the resultant thin films was studied using surface plasmon resonance [48-50] and ellipsometry. The subsequent growth of alternatively charged dendrimer multilayers shows uniform progress in film thickness. As a preliminary study for bimolecular interaction, a cell-culture study on foetal cortical neurons was performed using cationic dendrimer [51]. The results showed better proliferative potential as well as faster maturity.

Polyamidoamine- (PAMAM-) based dendrimers have also been used as a template to fabricate PAMAM-CdS nanocomposites, which show stable photoluminescence in various solvents, such as water and methanol [52-56]. By the application of methanolic Cd^2+^ and S^2−^, PAMAM-CdS nanocomposites with amine-terminated polyamidoamine dendrimers were synthesized [57]. Polyamidoamine is used extensively in various areas: medical [58], catalysis [59], fabrication of nanoparticles [60], etc. In one recently developed technique, composite microspheres were fabricated by alternately adsorbing polyelectrolytes and nanoparticles on latex beads by electrostatic interaction [61-63].

Polyamidoamines have positive as well as negative charges on their external surface, which facilitates adsorption of oppositely charged substrates and microspheres by electrostatic forces [64–65]. A spectrophotometric study of self-assembly processes shows that, as the number of layers of PAMAM-CdS nanocomposites on melamine formaldehyde (MF) microspheres increases, the resultant colour of melamine formaldehyde microspheres also changes, turning to yellow, confirming the layer-by-layer adsorption of PAMAM-CdS on the outer surface of MF microspheres. This study also confirms that photoluminescence intensity of the imprinted composite microspheres can be varied by adjusting layers of coated PAMAM-CdS nanocomposites on melamine formaldehyde (MF) microspheres [66].

### 2.4 Carbon-nanotubes-based thin film

Since the discovery of carbon nanotubes (CNTs), they have been often tried and used in the development of chemical sensors, stress/strain sensors, biological sensors, scanning probes and field-emission displays. Carbon nanotubes are also an important component of nano electromechanical systems (NEMS) and nano electronic devices [67-71]. One of the biggest limitations of CNT-based devices is the precise deposition of CNTs on a solid substrate. To overcome this limitation a controlled deposition of CNTs is required, which can be achieved by various approaches such as Langmuir-Blodgett, Chemical Vapour Deposition (CVD) and selective electrophoresis deposition [72, 73]. Layer-by-layer self-assembly is an effective as well as economic process to fabricate well-organized multilayers at nanometre scale. The biggest advantages of layer-by-layer self-assembled thin films are that we can deposit those thin films on the surface of almost any type of material with any topography. Thin films of CNTs formed by the layer-by-layer self-assembly approach show enhanced mechanical properties when compared to CNT/polymer matrices [74]. Various studies pertaining to the fabrication of two-and three-dimensional microstructures based on singlewalled carbon nanotubes (SWNT) multilayers. It demonstrates a ‘bottom-up’ approach to alternately deposit poly(dimethyldiallylammonium chloride) (PDDA) and SWNTs onto silicon and polymer substrates. The resultant single-walled-nanotube micro patterns were observed using scanning electron microscopy (SEM). Three-dimensional single-walled-carbon-nanotubes-based magnetic cantilever arrays were fabricated using poly (dimethyldiallylammonium chloride) (PDDA) and Fe_2_O_3_, and used to check potential applicability. A modified liftoff strategy was used to provide an additional safeguard for the cantilever structures.

The layer-by-layer self-assembly technique can also used to prepare enzymatic thin films. This electrostatic self-assembly (ESA) method is used for electrostatic adsorption of cationic and anionic polyelectrolytes on charged surfaces for the fabrication of organized ultrathin polymer films [75, 76]. Different types of enzyme (glucose oxidase, alcohol oxidase, cytochrome oxidase, fructose dehydrogenase, horseradish peroxidise, soybean peroxidise, polyphenol oxidase, cholesterol esterase, urease) are assembled with redox [77-86], non-redox [87-90], or conductive [91] polyelectrolytes in efficiently organized multilayers by electrostatic adsorption. Recent studies show the possibility to produce stable and reproducible enzymatic films by building polyelectrolyte enzyme assemblies onto glassy carbon surfaces [92].

Different types of techniques applied to functionalize the glassy carbon (GC) surfaces with a negatively charged layer of 4-phenylacetate groups are covalently graphed onto the glassy carbon surfaces to impart initial negative charge. In another approach, a glucose oxidase (GOD) monolayer is formed by an affinity reaction between a glucose-oxidase-conjugated antibody and adsorbed antigen monolayer. An immunologically active layer is developed on the substrate surface by the deposition of an antigen and antibody, leading to an antigen-antibody reaction.

These GCA- and GCB-coated glassy carbon surfaces, after washing, were dipped alternately into either poly(dimethyldiallylammonium) (PDDA) or poly(styrenesulphonate) (PSS) solutions formulated in saline buffer. Subsequently (GOD-PDDA)_n_ multilayers were assembled by dipping the GCA/PF and GCB/PF (PF-precursor films) alternately into glucose oxidase and poly(dimethyl-diallylammonium) solutions prepared in buffer saline. Studies suggest that immunology-based modifications of GC surfaces using a protein monolayer can establish a platform for different electrostatic-enzyme multilayer assemblies [93].

### 2.5 Formation of anti-reflection (AR) thin films

Anti-reflection (AR) thin films with layers of high and low refractive index are applied in various utilities such as optical materials, display devices, solar cells and glazings [94-103]. Principally, higher efficiency can be achieved by higher transmittance and decreasing the reflection. Different types of materials such as polymers [97], polyelectrolytes [104, 105], SiO_2_ [106, 107], TiO_2_[108, 109], SiO_2_/TiO_2_[110, 111] and Al-doped ZnO are used for fabrication of anti-reflective (AR) coatings. In recently adapted approaches the layer-by-layer self-assembly method is used for fabrication of anti-reflective coating films [112]. By application of the layer-by-layer approach, oppositely charged materials are deposited on the substrate by application of electrostatic forces, and hence formation of thin films [113] with multiple features take place. This layer-by-layer self-assembly approach can also be used to control the porosity and thickness of the multilayers [114]. pH is an important criterion to regulate the morphology and thickness of the AR-coating films [115, 116]. Atomic Force Microscopy is used to study the refractive index, roughness, bilayer thickness of poly(dimethyldiallylammonium chloride)/titanium(IV) bis(ammonium lactate) dihydroxide(TALH) as well as for poly(allylamine hydrochloride) and poly(acrylic acid).

### 2.6 Preparation of anti-UV multilayer coatings

An electrostatic self-assembly (ESA) technique can be used for protection of cotton fabrics from the harmful effects of UV radiation. There are several advantages associated with ESA processing, such as no constraints on size, shape and topography of the charged substrates to be utilized. Fictionalization can be controlled by applying altering polyelectrolyte solutions or charged substances by simple, eco-friendly and energy-preserving approaches. Various types of organic molecules with positive as well as negative charges can also be integrated by using multilayers via layer-by-layer self-assembly deposition [105-111].

Recently, using the ESA technique, anti-UV multilayer coatings on cationic cotton fabrics were developed. In this approach, three different types of fluorescent brightening agents (FBAs) incorporated with anionic and polyelectrolytes on cotton substrates were used in alternative layer-by-layer self-assembly [113-122]. The formed multilayer ultrathin films on cotton substrates were characterized by their durability and growth requirement. Layer-by-layer ESA deposition of fluorescent brightening agents and polycations (PDDAs) was used to achieve the anti-UV function of cationic cotton. The assembled cotton substrates were further characterized for surface polarity and multilayer growth through the colour-yields index of cotton surfaces [123-128].

Self-assembled thin films were also formed by sequential deposition of aqueous solution of squarylium (SQ) dye and poly(dimethyldiallylammonium chloride) (PDDA) onto a glass substrate. This is an effective example of ultrathin organic multilayer films [129-136]. Squarylium dyes and their derivatives are 1, 3-disubstituted compounds synthesised from squaric acid in the presence of two electrondonating aromatic or heterocyclic methylene bases. The squarylium dyes have utility as organic xerographic photoreceptors [137], as optics-based recording media [138], and organic solar-based cells [139]. These cyanine-based dyes have beneficial attributes such as photoconductivity, and intense and sharp absorption in the visible or infrared region [140-142].

## 3. Applications of layer-by-layer self-assembly

Layer-by-layer self-assemblies can be utilised in various devices for different functions.

### 3.1 Gas sensors

Titanium dioxide (TiO_2_) nanoparticles are used to develop sensors for measuring frequency shifts as a function of gas concentration and relative humidity [143]. Due to the higher surface area of these nanoparticles, they are used for their gas sensitivity, which can also be enhanced. A thin film of weak polyelectrolytes and TiO_2_ nanoparticles is produced by layer-by-layer self-assembly using oppositely charged solution on quartz-crystal microbalance (QCM). A subsequent acidic treatment given to these films is followed by neutral water treatment. This treatment usually breaks ionic bonds of weak electrolytes and thus separates the resultant aggregated TiO_2_ nanoparticles in the thin film. Other applications of TiO_2_ nanoparticles also include photo catalysts [144], smoke filters [145], air filters [146] and optical filters [147].

### 3.2 Dye-sensitized solar cells

Crystalline TiO_2_-nanoparticles-based films with high porosity were synthesized using spongy replica to fabricate dye-sensitized solar cells [148]. This fabricated porous TiO_2_ film can be used not only as photo electrode for dye-sensitized solar cells but can also facilitate improving the photocurrent-voltage characteristics. These organic multilayer thin films are formed on substrate by sequentially dipping alternately negatively charged polyanion as well as polycation [149]. Polymers such as poly(allylamine hydrochloride) (PAH) and poly(acrylic acid) (PAA) are used to form very thin film (∼1 μm) replicas. TiO_2_ is deposited on these porous polymer films. The fabrication of controlled porous TiO_2_ films is also achieved by removing polymers through calcination [150].

### 3.3 H2 sensing

There are multiple areas of application for H_2_ sensing, such as H_2_-incorporated engines and various industrial processes utilizing H_2_ gas [150, 151]. Polymers like polyaniline, polythiophene and polypyrrole are generally used in this technique due to their mechanical and electrical properties. They can be used in actuators, sensors and electro-chromic devices [152, 153]. To fabricate SWCNTs on polyester (PET) [154-156], dry-transfer printing technologies in conjunction with an electron-beam evaporation process are used to form thin layers of Pd. This strategy is used for decoration of SWCNTs with Pd nanoparticles. In-situ layer-by-layer self-assemblies of MWCNT-based thin films on PET substrate were fabricated using flexible H_2_ gas sensors and modified with Pd nanoparticles [157]. The layer-by-layer self-assemblies of polypyrrole (PPy) thin film on a PET substrate were modified by Pt nanoparticles and studied using Scanning Electron Microscopy (SEM) [158]. The in-situ self-assembled platinum-polypyrrole (Pt-PPy) thin film is produced from reduction of a Pt-based complex. Hence the resultant nanoparticle with improved sensitivity of polypyrrole-based thin films is achieved by rendering catalytically active sites to H_2_ gas molecules.

### 3.4 Stent-assisted gene transfer

Arterial diseases can be treated and in-stent restenosis can be prevented by intravascular stent-assisted gene transfer [159, 160]. A stent helps to facilitate local and efficient administration of therapeutic genes to the target cells of vascular wall. Polymers such as polylactic-polyglycolic acid copolymer (PLGA) [161], polyurethane [162], collagen (denatured) [163], polymers based on phosphorylcholine [164], and gelatine hydrogel [165] in conjunction with plasmid and adenovirus vectors are loaded onto the surface of the stent. For localized and prolonged availability of vectors, an efficient DNA uptake by cells and site of expression at vascular walls is needed. Recently stents have been designed by layer-by-layer self-assembly [166] and utilized for loading plasmids onto metal surfaces.

A lipid-plasmid complex (cationic), known as a cationic assembler, and a free plasmid acting as an anionic assembler were fabricated using layer-by-layer self-assembly to form a multilayer film with gold surface (acting as substrate) [167]. These self-assembled monolayers of carboxylic-acid-terminated alkanethiol (COOH-SAM) were modified and further characterized by water-contact angle measurements and Surface Plasmon Resonance spectroscopy. Gene-expression efficiencies were evaluated using Green Fluorescent Protein (GFP), seeding mammalian cells onto the multilayer surface loaded with a designed plasmid.

### 3.5 Prevention of phase separation of CNT-based composites

Different types of multilayers can be fabricated using cationic carbon nanotubes with anionic polyanions or anionic carbon nanotubes modified non-covalently with cationic naphthalenes on their outer walls [168]. A major limitations of this strategy is weak interaction between polyaromatic ionic molecules and nanotubes, leading to instability.

Electrostatic interaction has been employed to fabricate multi-walled carbon nanotubes (MWNT) multilayer films to construct the layer-by-layer self-assembly of anionic and cationic MWNTs [169]. The development pattern of multi-walled carbon nanotubes' layered structure is uniform, which allows the control fabrication of the multilayers and also prevents their phase separation in carbon-nanotube-/polymer-based composite films.

### 3.6 Nanoparticle coatings on fibres

Multilayers of TiO_2_ or SiO_2_ spherical nanoparticles and halloysite nanotubes were deposited on Kraft softwood fibres through layer-by-layer nano assembly, using them alternately with oppositely charged polyelectrolyte solutions [170, 171]. This method of deposition reasonably diminishes the possibility of hydrogen-bond formation at the contact region between neighbouring fibres. Layer-by-layer nano assemblies of different nanoparticles such as silica and TiO_2_ nanoparticles, clays such as montmorillonite and tubular halloysite nanotubes were developed on different supports using one or more layers of these nanoparticles by keeping them together through polyion interlayers. Kraft fibres are usually negatively charged [172-175]. These fibres have a complicated structure made by twisting high-molecular-weight cellulose polymers. Hydrogen bonding between cellulose fibres lends strength to paper during paper manufacturing and drying. Addition of silica, TiO_2_, clay minerals and other micro/nano particles is required to provide the necessary opaqueness, brightness and wettability to paper.

### 3.7 Formation of DNA-dye complex films

A preferred path for electron transfer is the stacking and overlapping of the n- and pi-electrons of DNA bases [176]. Biological sensors can be developed from thin organic films in which DNA is oriented and/or embedded [177]. The sequential deposition of 5, 10, 15, 20-tetrakis (4-N-methyl-pyridyl) porphine tetra(*p*-toluenesulphonate) (TMPyP) and deoxyribonucleic acid from aqueous solution onto quartz substrates results in the formation of complex films [178]. TMPyP can either bind with DNA electrostatically or by intercalating within the base pairs. In the formation of DNA-dye film, the resultant charge on the dye is important. These DNA-based films are assembled by using the layer-by-layer method by combining DNA with poly(allylamine hydrochloride) (PAH) [179] or Zr(IV) ions [180]. Despite interaction of DNA with dyes they retain their conformation as long as they can interact with different dyes in fabricated films.

### 3.8 Silver nanoparticles capped with mercaptosulphonic acid:

Surface-enhanced Raman spectroscopy (SERS) is a powerful micro-analytical technique used in various fields such as biomedicine [181], thin-film characterization [182], and trace-residue analysis [183]. For the fabrication of nanostructure layer-by-layer films, metal nanoparticles are used as functionally active building blocks. Thin films formed by this approach could be used for extending the selectivity of the matrix of SERS studies. Silver colloids exhibited superior catalytic activity [184] and more dependable enhancement elements for SERS [185]. Silver colloids have been synthesized by various methods, such as chemical reduction [186-189], photochemical methods [190], gamma irradiation [191], and laser ablation of bulk silver surfaces [192]. Of these methods, the chemical-based reduction approach is used extensively. Reducing agents like citrate, ethylenediaminetetraacetic acid, dye molecules and NaBH_4_ can be used. Mercaptosulphonic acid can be employed as stabilizer for the preparation of silver nanoparticles [193]. The resultant nanoparticles show a negative charge in aqueous solution. By the application of layer-by-layer self-assembly-based electrostatic interactions, these silver nanoparticles were can be transferred onto quartz slides to serve as active substrates in surface-enhanced Raman scattering.

### 3.9 Formation of Yb2O3-SiO2 coating microstructures

In the fabrication of layer-by-layer self-assembly, thickness and uniformity of multilayer particle assembly can only be controlled by depositing one particle layer at a time and then passing a complex-shape substrate through cellular and micro reactors [194, 195]. Multilayer particle assemblies that comprise Yb_2_O_3_ and SiO_2_ particles can be fabricated on Si support using layer-by-layer self-assembly. This is a new avenue to form uniform and dense Yb_2_O_3_-SiO_2_-coating microstructures. Basically, assemblies of both Yb_2_O_3_ and SiO_2_ particles require application of viscous-flow sintering for fabrication [196]. This consolidates and densifies the multilayer assemblies. A Yb_2_O_3_-SiO_2_ system can be selected due to its role in explicating and developing environmental barrier coatings (EBCs) for various Si-based materials [197, 198]. Yb_2_SiO_5_ and Yb_2_Si_2_O_7_ can be usefully used as coating materials for protection of Si_3_N_4_ ceramics in high-temperature turbine environments [199]. Various types of coating methods, such as electron beam, physical vapour deposition and air plasma spray have been used. Chemical vapour deposition can be used for dense coating structures but control of compositional uniformity of multicomponent coatings might pose a problem [200]. Slurry-based methods and sol-gel methods do not form a coating with uniform thickness or coverage control, though they offer low cost [201].

### 3.10. Solubility enhancement of CNTs in water

Drop-coating technology is used for formation of composite films. These formed films can be used for their ability to promote the electrochemical behaviour of biological as well as environmentally crucial compounds [202]. The limitations associated with these films relate to their stability and uniformity, which become compromised. This can be overcome by immobilization of carbon nanotubes on polymers, such that the carbon-nanotubes-based composite films enhance stability and uniformity. Recently, carbon-nanotubes-based biosensors were also fabricated by immobilizing biological molecules. Principally, different types of nanoelectronic devices can be developed by arranging carbon nanotubes appropriately [203]. One limitation associated with CNTs is their poor solubility, which constrains their applicability. The solubility of CNTs can be improved by grafting oxygen-containing groups at the side wall or ends of CNTs [204]. It can also be enhanced by covalent modifications, which include modification of carbon nanotubes with soluble compounds such as glucosamine [205]. In one newly developed approach, CNTs were modified with polycation poly(dimethyldial-lylammonium) (PDDA) and this stable complex was subjugated to layer-by-layer self-assembly using polyanion polystyrene sulphonate; the resultant electro-catalysis of the film to NADH was then analysed [205].

### 3.11. Improving the strength and durability of multilayer films for MEMS

Micro and Nano Electro Mechanical Systems (MEMS/NEMS) represent a turbulent interdisciplinary field that can find application in multiple components in variable portable devices. Incorporation of protective coating is crucial in micro electromechanical systems (MEMS) and nano electromechanical systems (NEMS) to give lower friction and higher anti-wear strength to assure the resultant performance, efficiency and dependability of devices [206]. Langmuir-Blodgett (LB) films are self-assembled monolayers and polyelectrolyte multilayers (PEMs), and serve as these protective coatings [207].

To impart specific properties, multiple components can be incorporated in nanocomposite films through layer-by-layer self-assembly, while for time- and cost-efficient fabrication of multilayer films, spin-assisted layer-by-layer self-assembly is used [208]. The introduction of SiO_2_ nanoparticles in multilayer films endows them with multifunctional features. In one strategy an ultra-hydrophobic surface was obtained by coating silica nanoparticles on a micro porous polyelectrolyte multilayer surface [209]. By using spin-assisted layer-by-layer self-assembly, SiO_2_ nanoparticles capped with Y-amino propyl trimethoxysilane were dipped into polyelectrolyte PAH and PAA multilayer films [210].

## 4. Conclusion

LBL self-assembly is an approach to develop an ultrathin film on solid support by alternate exposure to positive and negative species with impromptu deposition of the oppositely charged ions. This review has summarized various advancements in the development of the layer-by-layer self-assembly technique and its applications. Fabrication of layer-by-layer self-assembly requires various substrates such as metals, glass, and polymers. Each substrate has its own unique application in relation to layer-by-layer self-assembly. Various types of multilayers have been self-assembled by the layer-by-layer approach and used in construction of fuel cells, amperometric biosensors, dendrimer multilayers, thin films, anti-reflection thin films and anti-UV multilayers. Important applications of layer-by-layer self-assembly include gas sensors, e.g., H_2_ gas sensing, dye-sensitized solar cells, stent-assisted gene transfer. Recently developed applications include layer-by-layer self-assembly of anionic and cationic CNTs, nanoparticle coatings on microfibres, layer-by-layer self-assembly of DNA-dye complex films, coatings on nanoparticles and improvement of strength and durability of multilayer films for micro electromechanical systems (MEMS) and nano electromechanical systems (NEMS). With substantial advancements in science and technology and greater availability of information regarding layer-by-layer self-assembly strategies, more and more devices, such as biosensors and electromechanical devices, can be expected to be developed using this technique. More applications for layer-by-layer self-assembly will also be developed.
